# Exploring the Association Between Hidradenitis Suppurativa and Hyperthyroidism

**DOI:** 10.7759/cureus.68485

**Published:** 2024-09-02

**Authors:** Sunaina Addanki, Lisa Patel, Krina Patel, Deepesh Khanna

**Affiliations:** 1 Neurology/Cardiology, Nova Southeastern University Dr. Kiran C. Patel College of Osteopathic Medicine, Fort Lauderdale, USA; 2 Osteopathic Medicine, Nova Southeastern University Dr. Kiran C. Patel College of Osteopathic Medicine, Fort Lauderdale, USA; 3 Foundational Sciences, Nova Southeastern University Dr. Kiran C. Patel College of Osteopathic Medicine, Fort Lauderdale, USA

**Keywords:** graves' disease, apocrine glands, hypermetabolic state, pilosebaceous skin units, hidradenitis suppurativa complication, hidradenitis suppurativa risk factors, inflammatory disease, autoimmune disease, hyperthyroidism, hidradenitis suppurativa

## Abstract

Purpose: Hidradenitis suppurativa (HS) is an inflammatory disease affecting the pilosebaceous skin units and is linked to several autoimmune conditions. An area of exploration includes the connection between hyperthyroidism and HS. This study aims to investigate and establish the relationship between HS and hyperthyroidism.

Methods: The relationship between hyperthyroidism and HS was evaluated using data from the National Institute of Health (NIH) All of Us Researcher Program. A cross-sectional study was performed to assess the prevalence of HS in individuals with and without a history of hyperthyroidism matched by age ranges and health surveys. Relative risk and significance were determined by using standard statistical methods.

Results: A total of 407,333 patients were matched by health surveys and age ranges in the control and experimental groups. Among patients with a history of hyperthyroidism, the prevalence of HS was 1.40% compared to 0.38% in the control group. This difference was statistically significant (p < 0.0001 with an OR = 3.717, 95% CI 3.038-4.548).

Conclusion: This study demonstrates a statistically significant correlation between hyperthyroidism and increased prevalence of HS. These results justify the need for further research regarding hyperthyroidism's role in HS and the potential screening tools and lifestyle management techniques that may be prevalent for both conditions.

## Introduction

Hidradenitis suppurativa (HS) is an inflammatory disease with equivocal etiology. The estimated global prevalence of this disease is found to be between 0.00033% and 1.4%, with a higher prevalence of 0.7% to 1.2% in European and US populations [1]. HS classically affects the pilosebaceous units in the skin, leading to abscess formation, sinus tracts, and scarring [2]. Axillary, groin, and perianal areas of the body are most affected as they are rich in apocrine glands [3]. HS manifests with a clinical spectrum ranging from mild discomfort to severe pain, leaving a profound impact on the quality of life of many individuals. Several studies have come to link autoimmune conditions such as arthritis, inflammatory bowel disease, type-1 diabetes, and hypothyroidism (Hashimoto's disease) with HS [3,4].

One particular area of ambiguity that remains is the connection between hyperthyroidism and HS. The prevalence of hyperthyroidism in the United States is approximately 1.2% of the population [1]. Hyperthyroidism is a state of increased thyroid hormones triiodothyronine (T3) and thyroxine. The most common cause of a hyperthyroid state is seen in Graves' disease, an autoimmune condition characterized by antibodies against thyroid-stimulating hormone (TSH) receptors. Some other causes of the hyperthyroid state are toxic multinodular goiter, subacute thyroiditis, and TSH-secreting pituitary adenoma [2-4]. Common symptoms include palpitations, heat intolerance, diarrhea, and weight loss, which are manifestations of heightened thyroid hormone levels [4].

It is imperative to determine a correlation between HS and hyperthyroidism for potential implications for patient care and to gain mechanistic insights into these disorders. Therefore, the aim of this study is to investigate a correlation between the prevalence of HS and hyperthyroidism while establishing a comprehensive understanding of the two conditions.

## Materials and methods

The relationship between hyperthyroidism and HS was evaluated using baseline data from the NIH All of Us (AoU) Researcher Program. The AoU workbench, a cloud-based platform, provides approved researchers with access to analyze data. Data were analyzed using Systematized Nomenclature of Medicine (SNOMED) codes and electronic health record (EHR) measurements based on the AoU Data and Statistics Dissemination Policy; no group counts under 20 were disclosed. The workbench comprises data from over 1,089,000 participants aged 18 years or older, along with EHR data, surveys, systolic and diastolic blood pressure, heart rate, height, weight, BMI, and multiple additional physical measurements. Participants were allowed not to answer the questions as well. EHR data were linked to consented participants, and participant privacy was maintained through a series of data transformations. This study was approved by the AoU Institutional Review Board (IRB), which reviews protocol, informed consent, and other participant-facing materials. The IRB follows the regulations and guidance of the Office for Human Research Protections. Consent for each participant was obtained via an e-consent evaluation, followed by the primary e-consent and HIPAA authorizations. The workbench's user interface tools were utilized, including Cohort Builder for participant selection, the Dataset Builder for creating datasets to analyze, and Jupyter Notebooks for data analysis. The Notebooks used saved datasets and direct queries through Python 3 and R programming codes. The R version 4.0.3 (The R Foundation, Vienna, Austria) was used to conduct our analysis [5]. Consequently, MS Excel (Microsoft Corporation, Redmond, Washington) was used to formulate graphs and tables to display the 95% confidence intervals and prevalence of HS.

A retrospective cohort study was performed by collecting secondary data for individuals with and without hyperthyroidism. Individuals were matched by age ranges and health surveys. Four distinct groups were established by applying specific inclusion and exclusion criteria. These groups included participants who were matched by health surveys and age ranges grouped by the following criteria: Group 1 included hyperthyroidism, which included HS; Group 2 included hyperthyroidism and excluded HS; Group 3 included HS and excluded hyperthyroidism; and Group 4 excluded hyperthyroidism and excluded HS. The SNOMED codes for hyperthyroidism included hyperthyroidism, hyperthyroidism due to ectopic thyroid nodule, and hyperthyroidism due to ectopic thyroid tissue. The SNOMED codes for HS included hidradenitis suppurativa. The experimental group consisted of individuals with both hyperthyroidism and HS, while the control group consisted of individuals with HS but without hyperthyroidism. A chi-square analysis was performed using Prism Statistical software (GraphPad Software, Inc., La Jolla, California) to determine significance and relative risk. Secondary data analysis was performed using JMP Pro 16.0 and R 4.0.3 [5]. Relative risk and significance were determined by using standard statistical methods.

## Results

**Figure 1:**
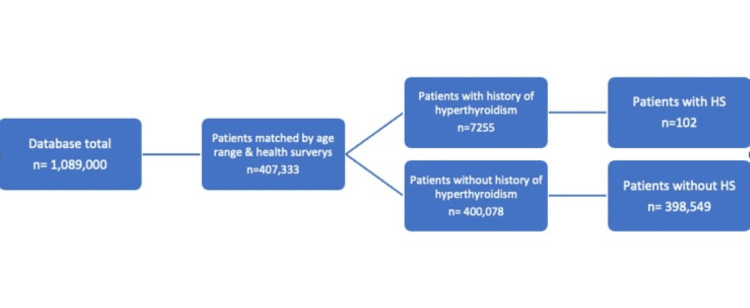
Diagram depicting the grouping of patients matched by age ranges and health surveys

Using the NIH AoU research database, 407,333 out of 1,089,000 participants were matched by age range and health surveys. The experimental group comprised 102 patients, whereas the control group included 1,529 patients (Figure 1).

**Figure 2:**
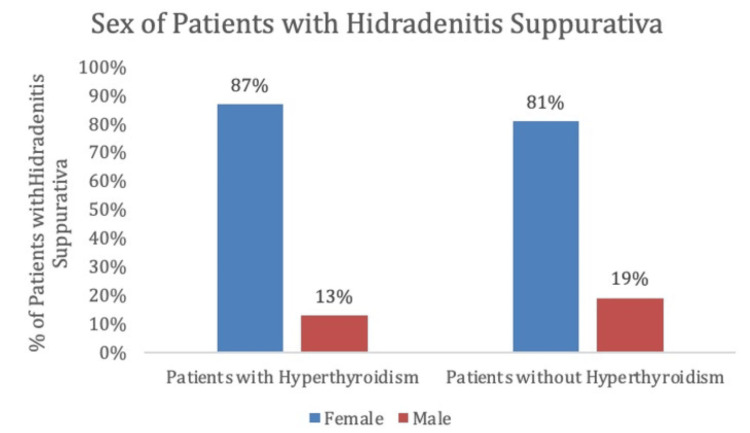
Percentages of patients with hidradenitis suppurativa with and without hyperthyroidism, matched by age ranges and health surveys

Among patients with a history of hyperthyroidism, the prevalence of HS was 1.40%, whereas the prevalence of HS was 0.38% in the control group. This disparity was statistically significant (p < 0.0001) with an odds ratio of 3.717 (95% CI 3.038-4.548) (Figure 2).

**Figure 3:**
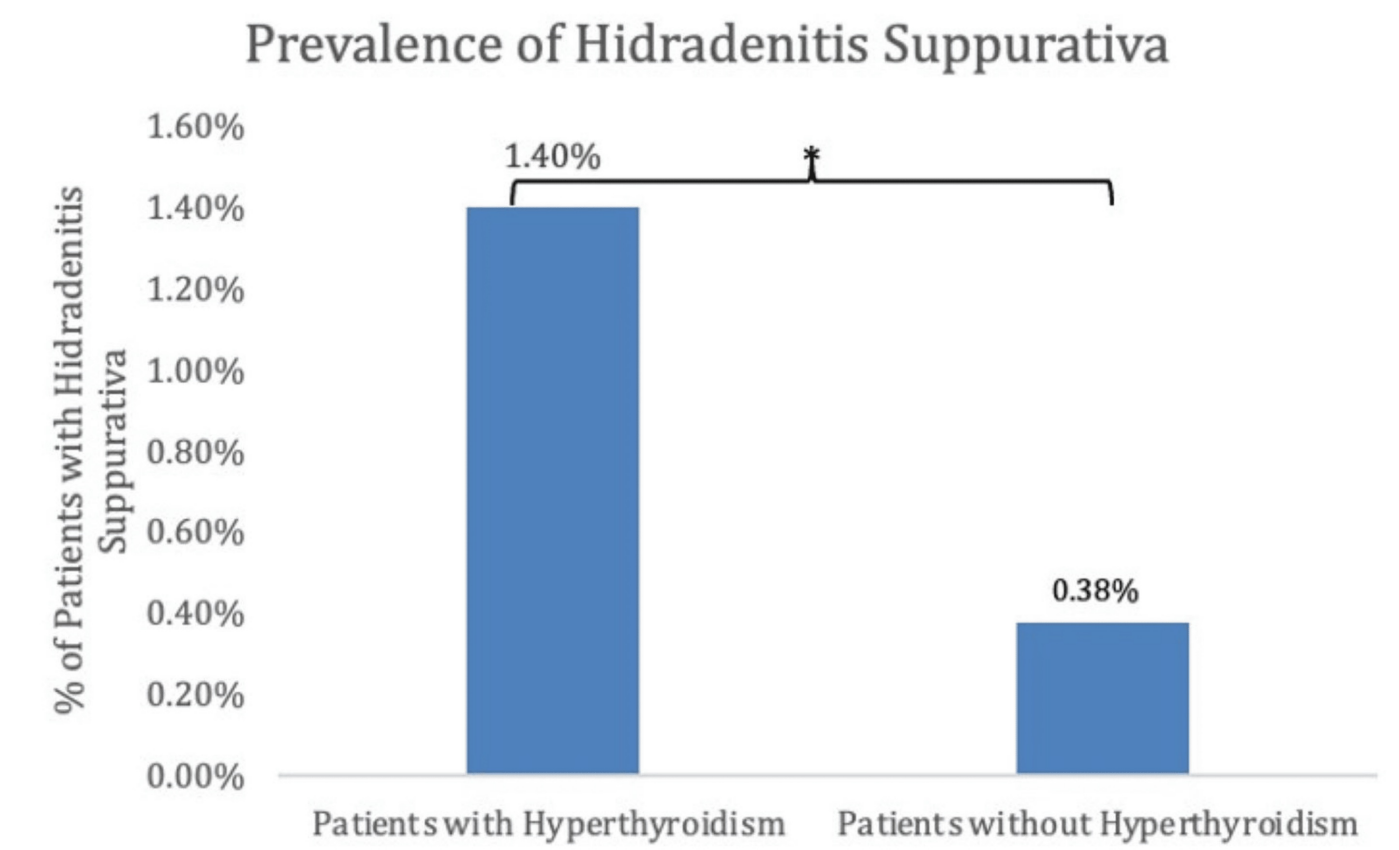
Percentages of patients with hidradenitis suppurativa with and without hyperthyroidism, matched by age ranges and health surveys

In terms of demographics, females comprised a greater proportion of patients with HS, both with and without a history of hyperthyroidism, accounting for 87% and 81% of the patient population, respectively (Table 1, Figure 3).

**Table 1:** Gender distribution of patients with hidradenitis suppurativa with and without a history of hyperthyroidism, matched by age ranges and health surveys

Sex of Patients With Hidradenitis Suppurativa		
Sex	Patients with hyperthyroidism	Patients without hyperthyroidism
Female	89 (87%)	1218 (81%)
Male	13 (13%)	280 (19%)

**Figure 4:**
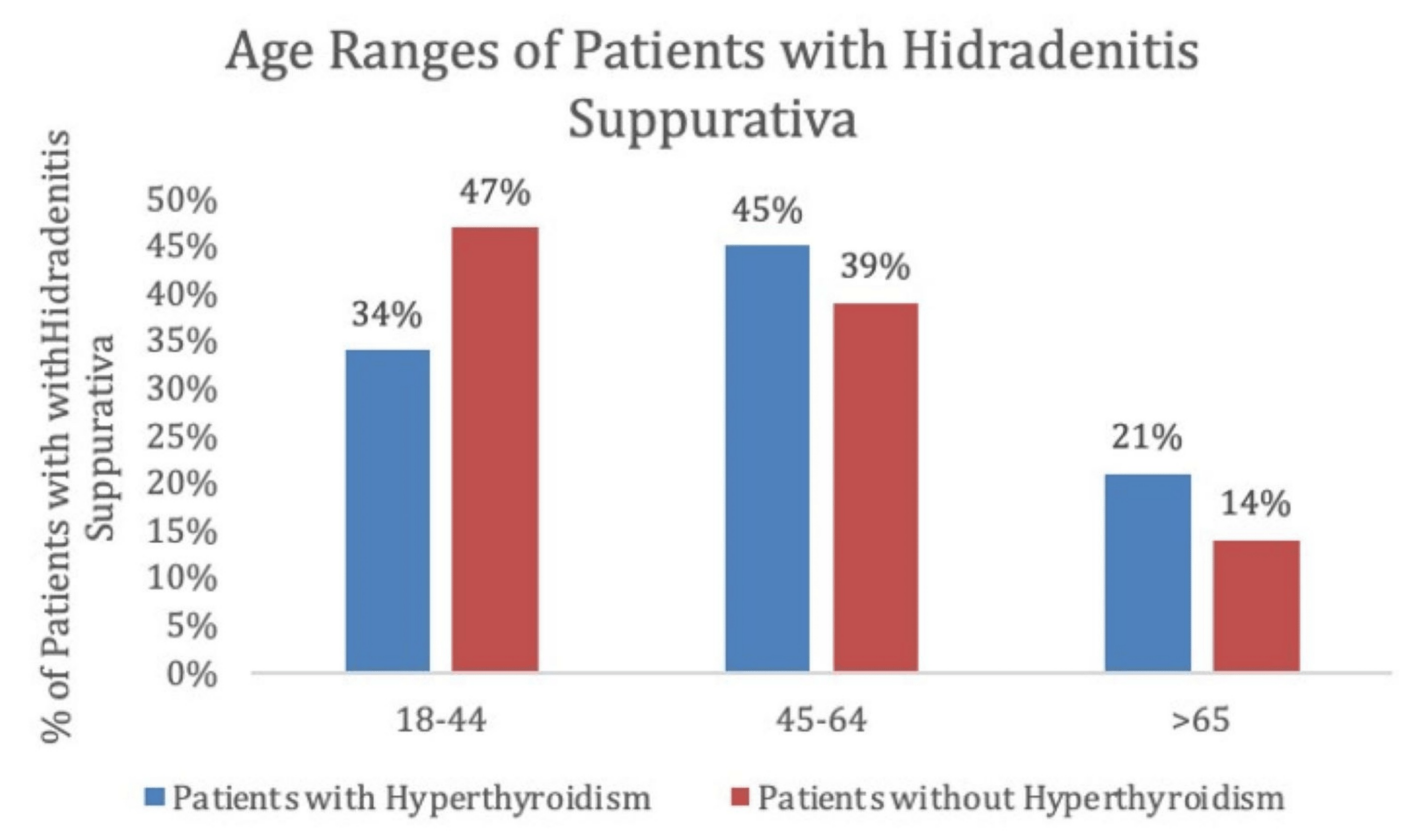
Percentage of patients with hidradenitis suppurativa with and without a history of hyperthyroidism at each age range, matched by age ranges and health surveys

When considering age ranges, in the control group, the largest percentage of patients was between the ages of 18 and 44, followed by 45-54, and >65 with 712 patients (47%), 607 patients (39%), and 123 patients (14%), respectively. In the experimental group, the largest percentage of patients were between the ages of 45 and 65, followed by 18-44, and >65 with 46 patients (45%), 35 patients (35%), and 21 patients (21%), respectively (Table 2, Figure 4).

**Table 2:** Age ranges of patients with hidradenitis suppurativa in patients with and without hyperthyroidism, matched by age ranges and health surveys

Age Ranges of Patients With Hidradenitis Suppurativa		
Age ranges	Patients with hyperthyroidism	Patients without hyperthyroidism
18-44	35 (34%)	712 (47%)
45-64	46 (45%)	607 (39%)
>65	21 (21%)	210 (14%)

**Figure 5:**
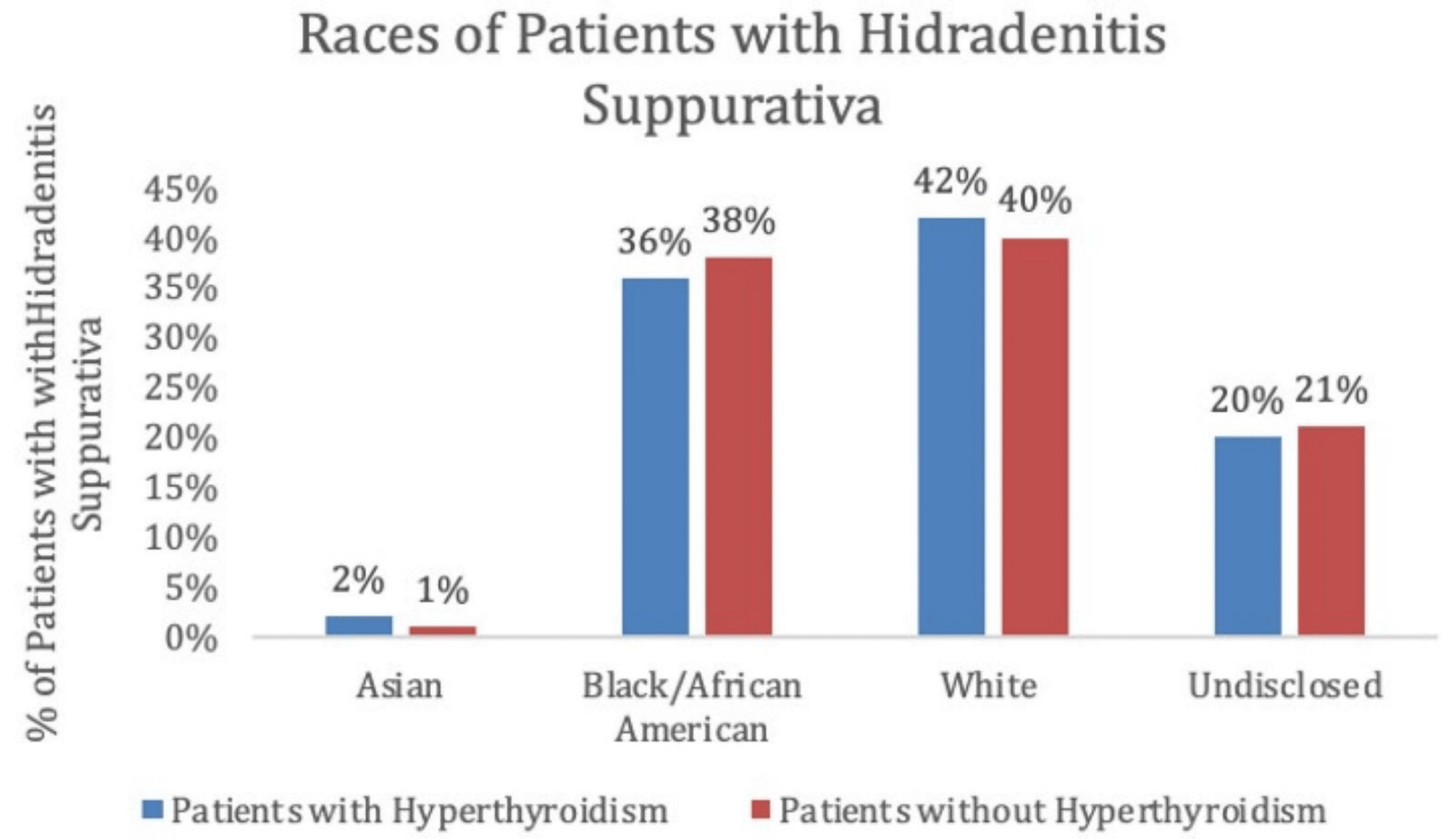
Percentage of patients with hidradenitis suppurativa with and without a history of hyperthyroidism for each race, matched by age ranges and health surveys

Finally, these data were analyzed to identify racial prevalence among the control and experimental groups. For both the experimental group and control group, White participants demonstrated the highest percentage of patients, 42% and 40%, respectively, while African American participants followed with 36% and 38%, respectively. The Asian population of participants was the lowest in both the experimental and control groups, with 2% and 1%, respectively. Approximately 20% of the experimental group and 21% of the control group remained undisclosed in terms of racial identification (Table 3, Figure 5).

**Table 3:** Races of patients with hidradenitis suppurativa with and without a history of hyperthyroidism, matched by age ranges and health surveys

Races of Patients With Hidradenitis Suppurativa		
Races	Patients with hyperthyroidism	Patients without hyperthyroidism
Asian	2 (2%)	15 (1%)
Black or African American	37 (36%)	575 (38%)
White	43 (42%)	612 (40%)
Nondisclosed	20 (20%)	327 (21%)

## Discussion

This retrospective cross-sectional study found a statistically significant difference between the prevalence of HS in those with hyperthyroidism compared to individuals without hyperthyroidism when matched by age ranges and health surveys. Our study showed that females accounted for the largest portion of patients with HS with and without hyperthyroidism. A study conducted by Sherman et al. also supported a significant correlation (p < 0.01) between females with HS with both hyperthyroidism and hypothyroidism [6]. Additionally, our data indicated that the largest population of individuals with hyperthyroidism and HS were between 45 and 64 years old. The previous study also corroborated these results, as they found a statistically significant correlation (p < 0.01 in ages 30-49 and p < 0.05 in ages 50-69) between age and TSH levels in those with HS [6]. Furthermore, our study showed that African American and White patients accounted for the majority of patients with HS and with or without hyperthyroidism. Studies have shown a higher prevalence of HS in African American individuals; however, no study could be found that correlated HS with hyperthyroidism among races [7]. However, there was no significant difference among races and age ranges in those with HS presenting with and without hyperthyroidism.

One possible explanation for the significance of HS in individuals with hyperthyroidism is metabolic hormone dysregulation. Hyperthyroidism is inherently a state of hyper-metabolic functioning where an excess of T3 hormone upregulates metabolic rate by binding nuclear receptors that increase transcription of Na-K+ ATPase. This accentuates oxygen consumption and heat production. Additionally, T3 binds adrenergic receptors to increase heart rate and glucose metabolism [8]. Previous studies have shown that obesity promotes an increase in TSH secretion due to decreased tissue responsiveness to thyroid hormones [9,10]. Thus, an increase in TSH and, subsequently, T3 is an attempt made by the body to stimulate ignorant tissue to respond to metabolic signals. This effectively combats excess weight by inducing a hypermetabolic state [10]. The propensity to have increased T3 in obese individuals may account for the significant correlation between HS and hyperthyroidism. Individuals with obesity are prone to the development of HS and disease progression due to increased intertriginous surface area [11]. These findings suggest that a significant correlation between these two conditions may be due to the metabolic hormone dysregulation commonly found in obesity. The severity of HS is another factor that can be attributed to disorganized metabolic hormones. In a state of excess T3, there is a rise in body temperature and thermogenesis, which exacerbates HS via excessive sweating and heat stress [12].

Although the mechanism of HS is not clear, another plausible explanation is the role of inflammatory markers TNF-α and IL-17. HS has been identified as a condition with elevated TNF-α and IL-17 levels [13]. TNF-α, along with IFN-γ, upregulates major histocompatibility complex-II (MHC-II) on thyroid epithelial cells that would normally be free of these receptors [13]. Increased aberrant expression of MHC-II can trigger thyroid autoimmunity and may explain a potential correlation between hyperthyroidism and those with HS. Furthermore, elevated TNF-α levels lead to hyperkeratinization of follicles and eventual rupture of abscesses seen in HS [14]. Another proposed mechanism involves IL-17. The Th17 pathway is upregulated in Graves' disease [6]. Th17 cells have been found to cause thyroid gland enlargement through independent mechanisms of TSH receptors. As Th17 cells produce IL-17, this cytokine is known to be an important regulator of cellular metabolism. Varying levels of these cytokines mediate metabolic adjustments and promote the proliferation of epithelial cells and skin stem cells. This marker also acts to maintain normal skin barrier functions [15]. However, elevated levels were seen in individuals with HS, increasing inflammation through unregulated keratinocyte activation [16]. The increased expression of these pathways in hyperthyroidism leads to compromised skin barrier function, which can be a possible explanation for the coexistence of HS in hyperthyroid states.

Our study is subject to several limitations. These results are based on the large database comprising patient laboratory data and electronic health records. EHRs are limited to single healthcare networks, indicating that out-of-network care was not included in this study. Additionally, the geographic focus of the AoU program tends to favor regions with larger recruitment funding, which may affect the representation of our data. In addition, we lack information on the completeness of data from each recruitment site within the program. Further research utilizing a prospective cohort approach may be beneficial to further substantiate the findings proposed in this paper. Applications of further research include generating early screening tools for HS and hyperthyroidism, implementing monitoring guidelines, and lifestyle management for both conditions. Additionally, future studies deciphering the specific causes of hyperthyroidism and the prevalence of HS among those conditions may assist in understanding pathophysiological processes and delivering more personalized treatment plans.

## Conclusions

This study concluded that there is a significant correlation between HS and hyperthyroidism. The pathogenesis of HS is still unclear but may be due to inflammatory markers, metabolic dysregulation, and increased thermogenesis. This study proves that there is merit in conducting further research into the relationship between HS and hyperthyroidism. It would be beneficial to conduct a prospective cohort study to determine the causality of these two conditions. Understanding the pathophysiological connections between these conditions may help develop more targeted and effective treatment strategies.
